# Individuals with FOXP1 syndrome present with a complex neurobehavioral profile with high rates of ADHD, anxiety, repetitive behaviors, and sensory symptoms

**DOI:** 10.1186/s13229-021-00469-z

**Published:** 2021-09-29

**Authors:** M. Pilar Trelles, Tess Levy, Bonnie Lerman, Paige Siper, Reymundo Lozano, Danielle Halpern, Hannah Walker, Jessica Zweifach, Yitzchak Frank, Jennifer Foss-Feig, Alexander Kolevzon, Joseph Buxbaum

**Affiliations:** 1 Seaver Autism Center for Research and Treatment, Icahn School of Medicine at Mount Sinai, New York, NY USA; 2 Department of Psychiatry, Icahn School of Medicine at Mount Sinai, New York, NY USA; 3 Mindich Child Health and Development Institute, Icahn School of Medicine at Mount Sinai, New York, NY USA; 4 Friedman Brain Institute, Icahn School of Medicine at Mount Sinai, New York, NY USA; 5 Department of Genetics and Genomic Sciences, Icahn School of Medicine at Mount Sinai, New York, NY USA; 6 Department of Pediatrics, Icahn School of Medicine at Mount Sinai, New York, NY USA

**Keywords:** FOXP1 gene, FOXP1 syndrome, Neurodevelopment, Intellectual disability, Attention-deficit/hyperactivity disorder, Autism spectrum disorder, Anxiety

## Abstract

**Background:**

FOXP1 syndrome is an autosomal dominant neurodevelopmental disorder characterized by intellectual disability, developmental delay, speech and language delays, and externalizing behaviors. We previously evaluated nine children and adolescents with FOXP1 syndrome to better characterize its phenotype. We identified specific areas of interest to be further explored, namely autism spectrum disorder (ASD) and internalizing and externalizing behaviors.

**Methods:**

Here, we assess a prospective cohort of additional 17 individuals to expand our initial analyses and focus on these areas of interest. An interdisciplinary group of clinicians evaluated neurodevelopmental, behavioral, and medical features in participants. We report results from this cohort both alone, and in combination with the previous cohort, where possible.

**Results:**

Previous observations of intellectual disability, motor delays, and language deficits were confirmed. In addition, 24% of the cohort met criteria for ASD. Seventy-five percent of individuals met DSM-5 criteria for attention-deficit/hyperactivity disorder and 38% for an anxiety disorder. Repetitive behaviors were almost universally present (95%) even without a diagnosis of ASD. Sensory symptoms, in particular sensory seeking, were common.

**Limitations:**

As FOXP1 syndrome is a rare disorder, sample size is limited.

**Conclusions:**

These findings have important implications for the treatment and care of individuals with FOXP1 syndrome. Notably, standardized testing for ASD showed high sensitivity, but low specificity, when compared to expert consensus diagnosis. Furthermore, many individuals in our cohort who received diagnoses of attention-deficit/hyperactivity disorder or anxiety disorder were not being treated for these symptoms; therefore, our findings suggest that there may be immediate areas for improvements in treatment for some individuals.

**Supplementary Information:**

The online version contains supplementary material available at 10.1186/s13229-021-00469-z.

## Background

Deep phenotypic characterization of individuals with functional disruptions in high-confidence autism spectrum disorder (ASD) and/or intellectual disability (ID) risk genes is a key first step in enhancing clinical care and advancing therapeutic development [1, 2]. Such approaches can lead to the identification of treatment targets and objective outcome measures.

*FOXP1* (OMIM 605515) is a highly conserved transcription factor of the forkhead box P (FOXP) subfamily of FOX transcription factors: mutations in this gene are associated with an autosomal dominant neurodevelopmental syndrome, first described over a decade ago [3–5]. Neurodevelopmental symptoms in FOXP1 syndrome were first attributed to *FOXP1* haploinsufficiency through the finding of full or partial deletions in affected individuals [3, 6]. Subsequently, loss of function and missense variants in *FOXP1* were described, confirming this relationship [7–11]. More recently, dominant negative missense variants have been implicated in the syndrome [10]. Individuals with FOXP1 syndrome (including individuals with mutations or deletion impacting the *FOXP1* gene) typically present with intellectual disability, speech and language deficits, hypotonia, features of ASD, and mild dysmorphic features [3, 7, 8, 11].

**Fig. 1:**
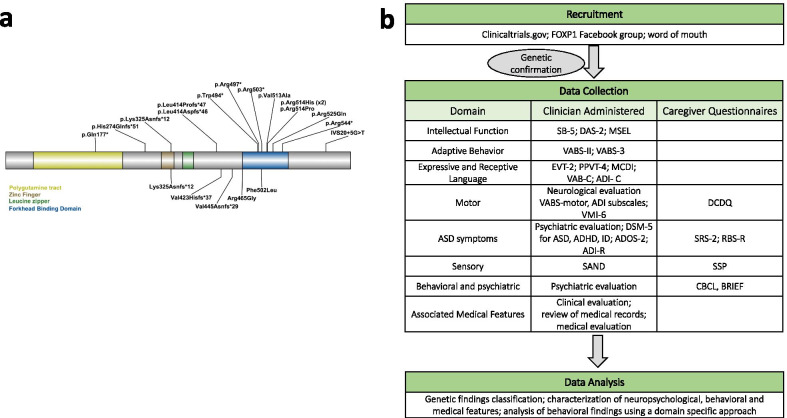
Assessment and methods. **a** Genetic landscape. **b** Assessment battery

FOXP proteins include a forkhead DNA-binding domain, a polyglutamine tract, a zinc finger domain, and a leucine zipper domain [12] (Fig. 1a). FOXP proteins can form homo- and hetero-dimers through the leucine zipper domain, and it is likely that various combinations of such dimers have differing impact on downstream targets [13, 14]. Interestingly, mutations in the gene FOXP2, which heterodimerizes with FOXP1, are associated with a rare speech and articulation disorder, with cerebellar involvement [15–19]. Pathogenic missense variants in *FOXP1* can result in the loss of ability to form dimers with FOXP2 [20, 21].

*FOXP1* is widely expressed in the developing and adult brain [12, 20, 22–24], and is considered a key regulatory gene during neural development [2, 25]. *FOXP1* has been implicated in transcription regulation mechanisms involved in neuronal migration and morphogenesis and synaptic plasticity. *FOXP1* knockdown mice exhibit impaired neonatal vocalizations related to atypical neural differentiation and cell migration during corticogenesis [25, 26]*.* FOXP1-regulated pathways have been implicated in striatal development [27], hippocampal function [2], neural-glial communication [28], and differentiation of dopamine neurons in the midbrain (Konstantoulas et al., 2010).

To date, more than 100 cases of FOXP1 syndrome have been described in the literature. Individuals present with delays in major developmental milestones often associated with mild to moderate intellectual disability [11, 29]. Children and adults with FOXP1 syndrome present with behavioral symptoms that often include externalizing behaviors (i.e., hyperactivity or impulsivity), anxiety, and autistic traits. FOXP1 syndrome is also associated with medical problems such as heart defects (22–40%), recurrent infections (e.g., sino-respiratory infections, skin infections) (33–67%), genitourinary abnormalities (13–42%), gastrointestinal problems (44–73%), and ocular defects (56–75%) [11, 29, 30]. Neurological abnormalities can include dysarthria, hypotonia, gait problems, feeding issues, atypical neuroimaging findings, and seizures [11, 29]. The present study aims to expand upon the existing literature by contributing results from systematic, comprehensive in-person evaluations of neuropsychological, behavioral, and medical features in individuals with FOXP1 syndrome using a prospective design and standardized measures.

Our group previously reported on a prospective cohort of nine children and adolescents with FOXP1 syndrome, five of whom were evaluated at our center [11]. This analysis provided initial characterization of the neuropsychological profile of the syndrome, while delineating associated behavioral and medical features. We noted mild to moderate impairment in cognitive and adaptive function, with associated delays in early motor and language milestones, language impairments, and deficits in visual-motor integration, as well as ASD in 25% of the participants—with a greater fraction endorsing ASD symptoms. We also noted high rates of attentional deficits, anxiety, externalizing symptoms, and obsessive behaviors. Importantly, the nature of these behavioral symptoms and their relationship to psychiatric diagnostic categories has not been adequately characterized and remains an important gap in the literature. For example, emotion dysregulation and irritability can be part of the symptom presentation of attention-deficit/hyperactivity disorder (ADHD) and of anxiety; nonetheless, treatment approaches would differ. Hence clarifying the nature of these symptoms and their relationship with psychiatric diagnostic categories stands to have important therapeutic implications for individuals with FOXP1 syndrome.

Here we report on 17 additional individuals, replicating findings from the first study and extending our analyses to areas of interest identified in the previous cohort. Specifically, we systematically characterize externalizing and internalizing behaviors and report on rates of ADHD symptoms (i.e., attentional deficits, hyperactivity, and impulsivity), as well as anxiety and externalizing symptoms such as irritability and aggression. Furthermore, we examined ASD symptomology in greater depth, including a more detailed analysis of repetitive behaviors and sensory symptoms, and we evaluate the utility of standardized diagnostic instruments in this population.

## Methods

### Participants

Seventeen individuals with FOXP1 syndrome (11 female, 6 male) between the ages of 2–33 years (9.24 ± 7.33) were enrolled between October 2016 and February 2020 (cohort 2). Study procedures were approved by the Program for the Protection of Human Subjects at Mount Sinai, and informed consent was obtained from the parents or legal guardians for all study participants. Participants assented when applicable. Subjects were recruited through word of mouth and via clinicaltrials.gov (Identifier: NCT03718923). Genetic testing was completed at a Clinical Laboratory Improvement Amendments (CLIA) certified laboratory; all variants were classified as pathogenic or likely pathogenic. Variants were annotated following the Human Genome Variation Society (HGVS) guidelines. FOXP1 RefSeq mRNA transcript and protein sequence, NM_032682.5 and NP_116071.2, were used to annotate cDNA and amino acid positions; deletions followed the human genome reference assembly Hg19 [GRCh37]. Where possible, data for the 17 participants (“cohort 2”) were combined with results from the 5 individuals previously seen at the Seaver Center (“cohort 1”) (combined *n* = 22) (Additional file 1: Table S1), although we also present results for the new cohort alone. In the combined cohort, participants identified as Caucasian (*n* = 18; 81%), Asian (*n* = 3; 14%), or Hispanic (*n* = 1; 5%).

### Assessment

Neurobehavioral and medical features were ascertained by an interdisciplinary group of clinicians (Fig. 1b).

#### Intellectual and adaptive functioning

Cognitive ability was measured using the Stanford Binet Intelligence Scales, Fifth Edition (Stanford Binet 5) [31], the Mullen Scales of Early Learning (Mullen Scales) [32], or the Differential Ability Scales—Second Edition (DAS-II) [33]. Tests were selected based on chronological age and language ability. Adaptive behavior was measured using the Vineland Adaptive Behavior Scales, Third Edition, Survey Interview Form (Vineland-3) [34, 35]. In cohort 1, the Vineland Adaptive Behavior Scales, Second Edition (VABS-II) (Sara S Sparrow et al., 1984) was used. Given normative differences, scores were not compared [36].

#### Language and communication

The Peabody Picture Vocabulary Test, 4th Edition (PPVT-4) [37] and the Expressive Vocabulary Test, 2nd Edition (EVT-2) [38] were used to test receptive and expressive language skills, respectively. In addition, the MacArthur–Bates Communicative Development Inventories [39] and Vineland Communication subdomains were used to assess language abilities.

#### Motor functioning

Motor skills were assessed through the relevant subdomains from the Vineland-3, the Beery–Buktenica Developmental Test of Visual-Motor Integration, Sixth Edition (VMI-6) [40], and the Developmental Coordination Disorder Questionnaire 2007 (DCDQ) [41]. Raw scores were used to obtain best estimates of motor function when standardized scores were not available.

#### Autism symptomatology and psychiatric comorbidities

Behavioral symptoms were assessed through (1) comprehensive psychiatric evaluation, (2) behavioral observations, and (3) administration of empirical instruments. Following the evaluation, all clinicians involved in the assessment (at least three) met to assign a Diagnostic and Statistical Manual for Mental Disorders Fifth Edition (DSM-5) [42] consensus diagnosis for ASD and ADHD and to characterize the presence or absence of anxiety and/or aggression during the evaluation. Anxiety and aggression were only considered significant if both the caregiver identified it as a chief concern and two independent clinicians observed and documented these behaviors during the evaluation (e.g., observed behavior in the ADOS and during the psychiatric evaluation). The administration of the assessment battery occurred over two consecutive days and lasted on average 10–12 h. One participant was excluded from this analysis given their developmental age at the time of the study.

1. The psychiatric evaluation was conducted by a child and adolescent psychiatrist with expertise in neurodevelopmental disorders and included a caregiver interview and direct assessment of the participant. The evaluation placed emphasis on the developmental history, and the assessment of externalizing behaviors, anxiety, compulsive behaviors, and ASD symptomatology (Table 1). Evaluations lasted on average 3 h.
2. Behavioral observations were documented during the administration of the assessment battery. The ADOS-2 systematically collects information on “Other Abnormal Behaviors” (E codes): overactivity (E1); tantrums, aggression and negative or disruptive behaviors (E2), and anxiety (E3). Only scores of 2 or 3 were considered problematic. In addition, observation of behavioral symptoms during the administration of other clinician-administered assessments (e.g., IQ, PPVT, or EVT) were documented for all participants. Examples of behaviors considered problematic include: “significant anxiety in response to requests,” “constantly moves,” “unable to settle for evaluation,” “hitting,” or “biting.” Behaviors were interpreted in the clinical context of the evaluation.
3. Empirical instruments (i.e., clinician-administered and caregiver questionnaires) were used to assess behavioral symptoms. ASD symptomatology was assessed using autism-specific measures, including the Autism Diagnostic Observation Schedule, Second Edition (ADOS-2) [43], and the Autism Diagnostic Interview—Revised (ADI-R) [44] were administered by research reliable clinicians. We used ADOS-2 social affect (SA) and repetitive behavior (RRB) calibrated scores to compare symptom domains across modules [45]. In addition, the Repetitive Behavior Scale—Revised (RBS-R) [46] was used to characterize repetitive behaviors, and the Social Responsiveness Scale, 2nd edition (SRS-2) [47, 48] was collected to measure social impairment. Sensory symptoms were measured using the Sensory Assessment for Neurodevelopmental Disorders (SAND) [49, 50] and the Short Sensory Profile (SSP) [51]. Furthermore, caregivers completed the Achenbach Child and Adult Behavior Checklist (CBCL 1 1/2–5 and 6–18, ABCL, respectively) [52]. All caregivers documented their three main concerns for their child as part of the CBCL/ABCL questionnaire. Examples of common concerns included “poor communication skills,” “anxiety or fears” “aggression” and “problems with attention.” In addition, executive function was assessed with the Behavior Rating Inventory of Executive Function (BRIEF-2) [53], a rating scale used to assess executive function in everyday behaviors.

**Table 1:** Background information

Psychiatric history (*n* = 21)	Prior/community	Current/consensus
DSM-5 diagnosis		
ASD	7 (33)	5 (24)
ADHD	7 (33)	16 (76)
Anxiety disorder	6 (29)	14 (67)
Psychotropic treatment (current)		
ADHD	4 (19)	
Anxiety	3 (15)	
Demographic (*N *= 22)	*M* (SD); *n*(%)	
Age		
Full sample	9.5 (6.7)	
< 5 years old	5 (23)	
5–11 years old	12 (54)	
11–18 years old	4 (18)	
> 18 years old	1 (5)	
Sex		
Female	11(65)	
Male	6 (35)	

One participant was excluded from psychiatric history analysis given their developmental age at the time of evaluation (< 18 months)

#### Medical evaluation

Comprehensive medical histories were collected by clinician interview of caregivers and medical records review. In addition, a clinical geneticist performed dysmorphology and physical exams. Semi-structured neurological evaluations were completed by a pediatric neurologist. Results from clinical electroencephalograms (EEGs) and brain magnetic resonance imaging (MRI) were reviewed when available, as was prior medical testing on organs of interest in the syndrome (e.g., echocardiograms, electrocardiograms, renal ultrasounds).

After completion of the evaluation, all participants received a clinical research summary detailing evaluation results.

### Data analysis

All analyses were performed using SPSS Statistics Version 24 [54]. Descriptive statistics were computed across all measures and phenotypic characteristics grouped and compared according to relevant domains. To facilitate graphical comparison, all variables were normalized and standardized into z scores by applying a Van der Waerden transformation. Scores for the RBS-R were Z-normalized against an ASD population for comparison [46]. A similar approach was used to analyze results from the SAND [50]. Finally, BRIEF scores were compared to previously published normed populations [53]. A detailed description of the populations used for comparison can be found in Lam and Aman [46] for the RBS-R and the manuals for the SAND and BRIEF. Exploratory analysis compared participants diagnosed with and without ASD, and/or ADHD for cognitive and adaptive functioning, language abilities, ASD and/or ADHD empirically measured symptoms. All comparisons were judged to be statistically significant at *p* < 0.05.

## Results

### Genetic findings

The combined cohort includes 22 individuals with pathogenic or likely pathogenic variants in FOXP1, of which 5 have been reported before (Fig. 1a). Two individuals carry deletions involving *FOXP1,* and the remaining 20 individuals carry sequence variants. One individual has a de novo deletion that includes approximately 30 genes, including the entirety of *FOXP1.* The deletion includes two other genes associated with autosomal dominant disorders: *PROK2* associated with hypogonadotropic hypogonadism 4 with or without anosmia (MIM 610628) and *ROBO2*, associated with vesicoureteral reflux 2 (MIM 610878). Individual 1477 carries a deletion that includes four exons of *FOXP1*. De novo status is unknown for this patient. Sequence variants included 7 missense, 6 frameshift, 5 nonsense, and 2 splice site variants. Variants were confirmed de novo in 18 individuals; in cases where the second parent was not available for testing, maternal inheritance was ruled out (*n* = 3). The cohort includes individuals with known recurrent variants, including p.Leu414Aspfs*26, p.Arg525Gln, p.Arg514His, and p.Arg503* (Additional file 2: Table S2).

### Neurobehavioral profile

#### Intellectual and adaptive functioning

**Fig. 2:**
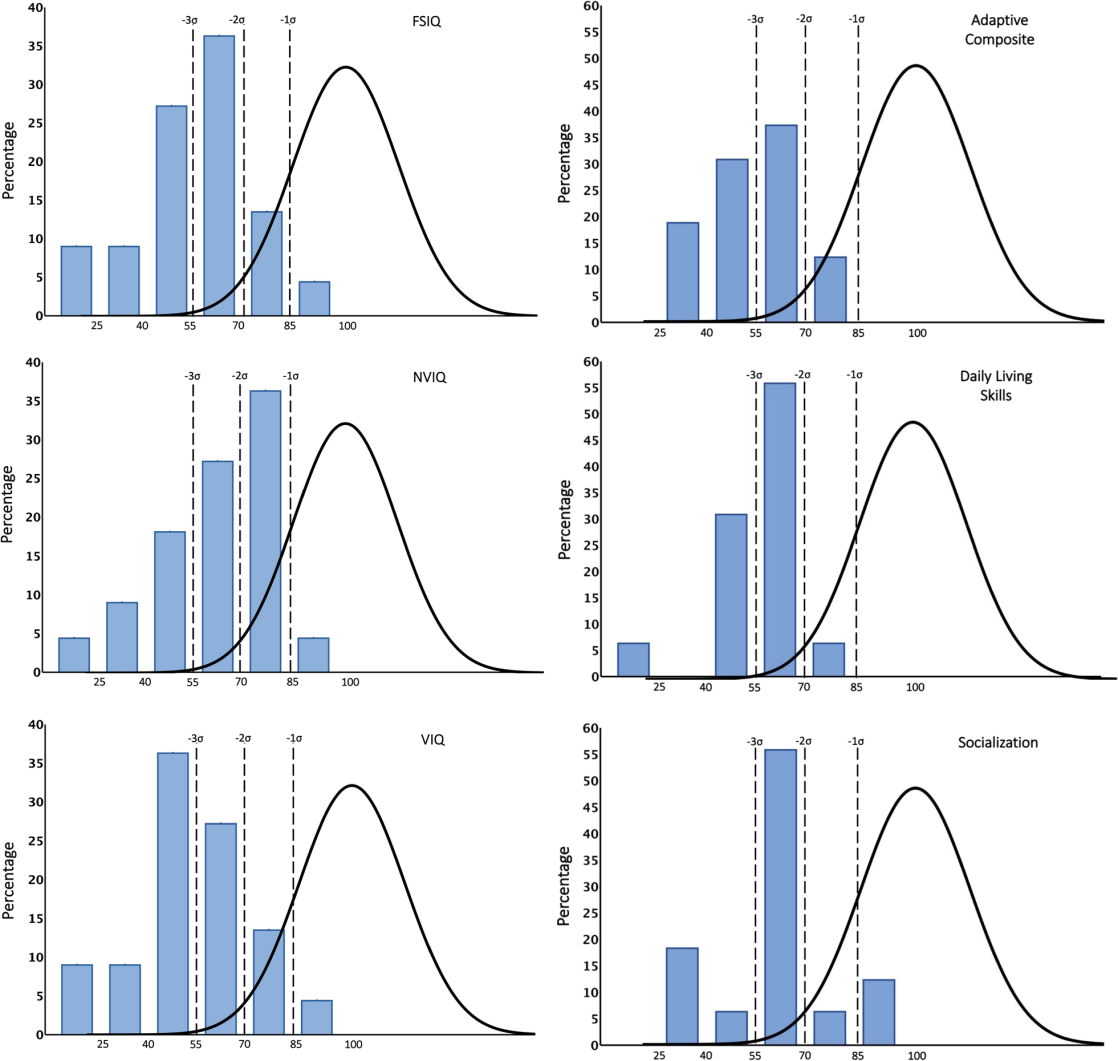
Intellectual and adaptive functioning. Intellectual quotient (IQ; *n* = 22) and adaptive function scores (Vineland-3; *n* = 16) are presented as standard scores, illustrating the distribution of scores as it compares to the general population

Intellectual quotient (IQ) scores (Fig. 2) were calculated for individuals who were administered the Stanford Binet 5 or the DAS-II. The Mullen Early Learning Composite was calculated for three of the five participants who were within the standardized age range (i.e., less than 68 months) [55]. For the remaining two participants, developmental quotients (DQ) were calculated and used for comparison purposes. Full scale IQ/DQ ranged from 14.6 to 93.0 (55.16 ± 18.94), with verbal abilities (verbal IQ/DQ = 54.42 ± 20.38) falling below nonverbal abilities (nonverbal IQ/DQ = 60.82 ± 19.38) for 77% of participants (*t*(16) = 5.68, *p* < 0.01). The Vineland-3 Adaptive Behavior Composite scores (Fig. 2) ranged from 32 to 80 (54.63 ± 13.86) and were comparable to IQ/DQ scores (*t*(15) = 0.086, *p* = 0.6). Socialization scores for participants with a DSM-5 diagnosis of ASD were lower than for participants without a diagnosis of ASD (*t*(14) = 1.77, *p* < 0.05). Scores for the Communication subdomain were significantly lower than Socialization (*t*(15) = 6.39, *p* < 0.01) and Daily Living scores (*t*(15) = 3.85, *p* < 0.01) across the sample. Figure 2 provides a graphical overview of score distributions for cognitive and adaptive function for the cohort in relation to the population norm.

#### Language and communication

**Fig. 3:**
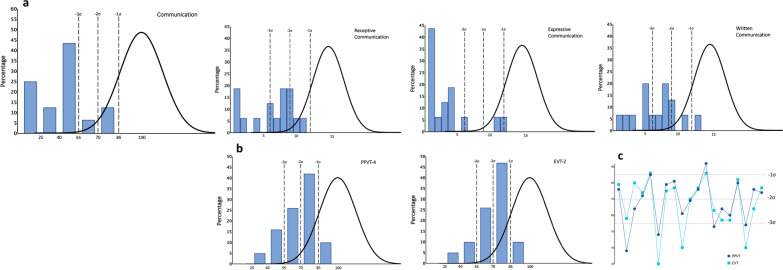
Language functioning. **a** Vineland Communication standard scores and subscale V scores (*n* = 16). **b** Receptive and expressive language are presented for comparison (*n* = 19). **c** PPVT and EVT standard scores presented for each participant. Two participants, with PPVT receptive standard score of 20, and who could not complete the EVT were excluded from figures

Individuals’ scores on the PPVT-4 and EVT-2 and Vineland-3 subscales were used to compare receptive and expressive language skills (Fig. 3). Results are graphically compared for the group and individual participants (Fig. 3b). Standard scores for the EVT-2 ranged from 30 to 86 and for the PPVT-4 ranged from 20 to 92. Two participants with standard scores of 20 on the PPVT-4 could not complete the EVT-2 and were excluded from analysis (Fig. 3c). PPVT-4 and EVT-2 scores were comparable (*t*(18) = 0.66, *p* = 0.51).

The Vineland-3 Communication domains yield subdomain scores and v-Scale scores (mean 15 and SD 3) for Receptive, Expressive, and Written language. v-Scale scores in the Receptive and Expressive subdomains ranged from 1 to 11 (6.25 ± 3.42) and 1 to 12 (3.50 ± 3.48), respectively. In the Written subdomain (*n* = 15), scores ranged from 1 to 13 (6.67 ± 3.29). Vineland-3 subdomains indicated that 9/16 (56%) had significantly higher receptive than expressive language abilities (*t*(8) = 8.66, *p* < 0.01).

The MacArthur–Bates Communicative Development Inventory was only collected on cohort 2, and participants exhibited a wide range of verbal abilities, with the number of Words Understood ranging from 7 to 396 (of 396) (310.12 ± 123.10) and the number of Words Produced ranging from 0 to 396 (of 396) (254.76 ± 159.02). Total Gestures ranged from 5 to 63 (43.59 ± 16.97), combining Early Gestures, which ranged from 5 to 18 (of 18) (13.71 ± 4.24), and Later Gestures, which ranged from 0 to 45 (of 45) (29.88 ± 13.31).

Language milestones were significantly delayed across the sample. The average age of first word and first phrase was 29 months and 44 months, respectively. The single participant in our cohort that did not use words at the time of evaluation was 25 months old.

#### Motor functioning

Motor milestones were assessed by the ADI-R. The age of first sitting ranged from 5 to 18 months (9.50 ± 3.11), age of first crawling ranged from 9 to 25 months (14.47 ± 54.56) where one individual (age 25 months at time of exam) had not yet reached this milestone, and age of first walking independently ranged from 13 to 38 months (21.75 ± 5.49). Again, the same individual had not achieved this milestone at the time of the evaluation.

Eleven of the 16 individuals who completed the Vineland-3 were administered the Motor Domain with standard scores ranging from 20 to 85 (53.09 ± 23.82). V-Scale scores in the Gross and Fine Motor subdomain were comparable (*t*(10) = 1.13, *p* = 0.28). Gross Motor scores ranged from 1 to 14 (6.45 ± 4.37), and Fine Motor scores ranged from 1 to 11 (5.55 ± 3.98).

The VMI-6 standard scores ranged from 43 to 85 (61.05 ± 15.23) where 7 individuals (33%) met the cutoff for the impaired range. Lastly, caregivers completed the DCDQ (score range 15–75), where all individuals received scores indicating the presence of a developmental coordination disorder (DCD). Total scores ranged from 15 to 57 (30.60 ± 10.69); subdomain scores in the Control During Movement domain ranged from 6 to 29 (14.00 ± 5.91), in the Fine Motor/Handwriting domain ranged from 4 to 10 (6.55 ± 2.14), and in General Coordination domain ranged from 5 to 19 (10.00 ± 3.95).

#### ASD symptomatology

**Fig. 4:**
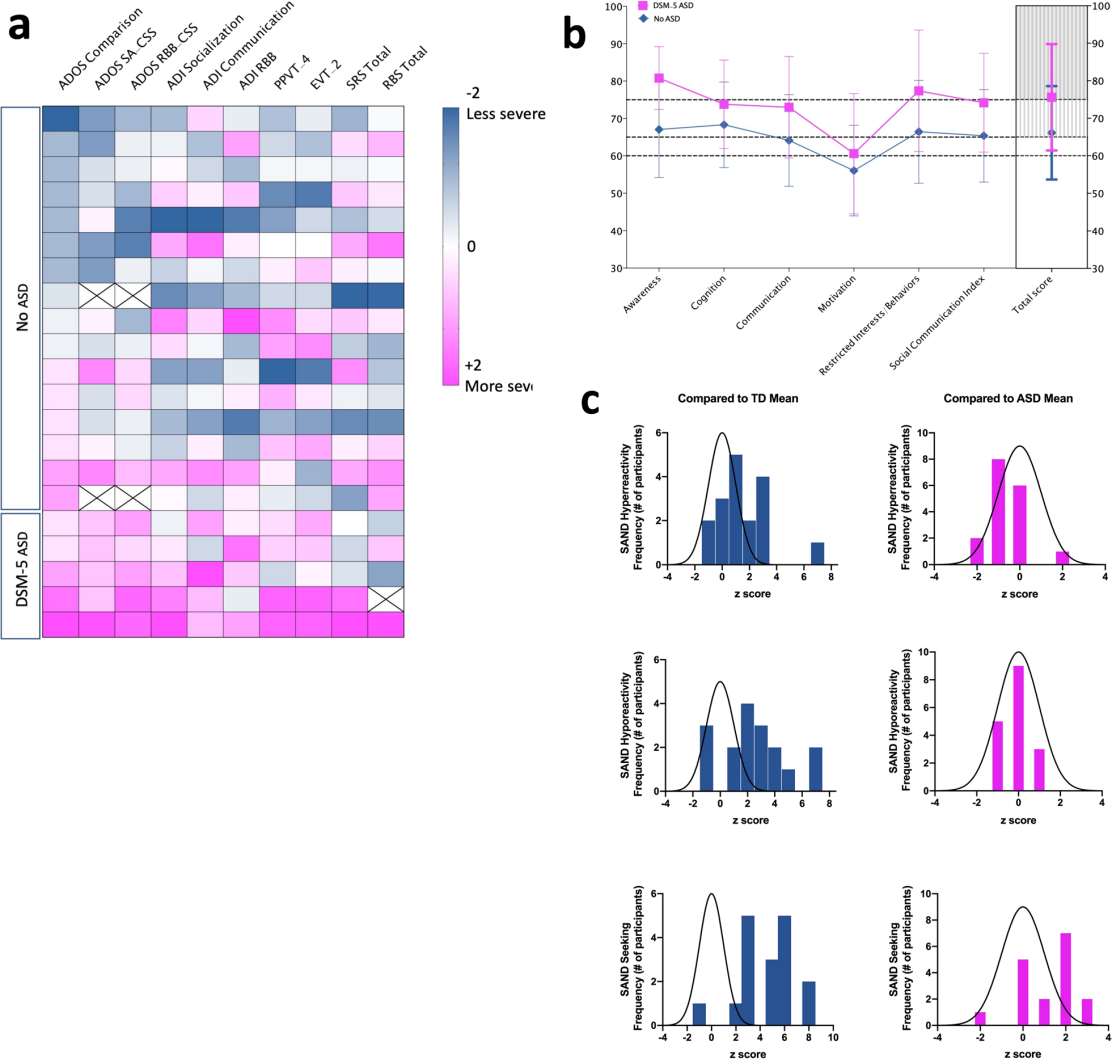
ASD symptomatology. **a** Z-normalized scores for each participant ordered by severity of ADOS-2 comparison scores from less severe to more severe. Each row represents a participant. Results show that despite a tendency toward more severe scores on instruments used to assess ASD for individuals with a DSM-5 ASD diagnosis, there is large variability in the neurobehavioral profile of individuals with FOXP1 syndrome. **b** Average SRS T-scores are presented comparing FOXP1 participants with and without ASD (bars represent standard deviation). **c** Sensory symptoms as assessed by the SAND are presented against a typically developing population and an ASD normed population

Forty-eight percent of participants (10/21) met criteria for autism, and 10% (2/21) for autism spectrum on the ADOS-2. In regard to the ADI-R, 47% (10/21) of participants were above the cutoff scores in all domains, 61% (13/21) met the cutoff for the Social domain, 76% (16/21) met the cutoff for the Communication domain, and (71%) 15/21 met the cutoff for the Restricted and Repetitive Behavior domain. Nonetheless, only 24% (5/21) of participants received a diagnosis of ASD based on expert clinical consensus (Fig. 4) as most did not meet DSM-5 criterion A for ASD (persistent deficits in social communication and social interaction across multiple contexts). For example, only five participants were deemed to have deficits in nonverbal communication. A detailed description of findings is presented in Additional file 6: Table S6. A diagnosis of ASD was deferred at the time of evaluation for one participant due to developmental age (i.e., less than 18 months). Four out of the five participants that met a consensus diagnosis of ASD were females. However, the proportion of females with and without a diagnosis of ASD was comparable [*X*^2^ (1, *n* = 21) = 0.24; *p* = 0.62].

The SRS-2 had average total T-scores falling in the moderate impairment range (68.18 ± 13.26). Average subdomain scores fell in the moderate impairment range in four of the five subscales, specifically, Social Awareness (70.14 ± 13.03), Social Cognition (69.95 ± 11.61), Social Communication (65.95 ± 12.94), and Restricted and Repetitive Behavior (69.18 ± 14.86). Interestingly, average scores for the Social Motivation subscale were in the typical range (56.50 ± 13.37). When individual total scores were analyzed, 31% (7/22) of participants scored in the severe range, 27% (6/22) of participants scored in the moderate range, 14% (3/22) of participants in the mild range, and 27% (6/22) in the typical range. As expected, mean SRS-2 scores across domains for participants with a consensus diagnosis of ASD had a tendency toward greater severity than those of individuals without ASD (Fig. 4b).

Caregivers completed the RBS-R. Individual total scores ranged from 0 to 62 (19.13 ± 16.22). To facilitate graphical comparison to the ASD population, RBS-R scores were Z-normalized against ASD normative data [46]. The presence of repetitive behaviors, as measured by the RBS-R (*t*(19) = 0.91, *p* = 0.37) or the restrictive and repetitive behavior subscale of the SRS-2 (*t*(19) = 1.44, *p* = 0.16), did not differ between those with and without a clinical diagnosis of ASD.

The SAND was administered to all participants and compared to age-matched typically developing (TD) controls (*n* = 54) (Fig. 4c). A detailed description of the characteristics of the comparison sample can be found in SAND manual [50]. The FOXP1 group displayed significantly more sensory symptoms compared to the TD group across symptom domains (hyperreactivity, hyporeactivity, seeking) and sensory modalities (visual, tactile, auditory). On average, within the FOXP1 group, sensory seeking (14.95 ± 7.42) was more common than hyperreactivity (6.09 ± 4.00) and hyporeactivity (5.86 ± 4.17) symptoms. Individuals with FOXP1 syndrome also showed a greater number of tactile symptoms (10.95 ± 4.98) as compared to auditory (8.36 ± 3.51) and visual (7.59 ± 4.90) symptoms. An analysis of subdomains indicated high levels of sensory seeking across visual, tactile, and auditory domains. High levels of tactile hyporeactivity (e.g., high pain threshold) and auditory hyperreactivity were also identified.

### Behavioral and psychiatric comorbidities

**Fig. 5:**
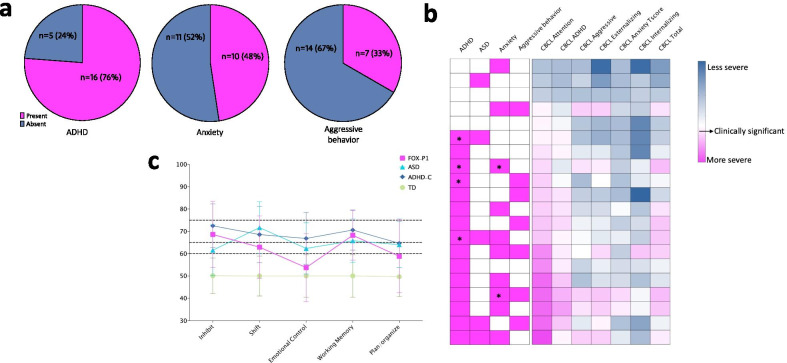
Behavioral and psychiatric profile. **a**. Participants who received ADHD and anxiety diagnoses, and those with a history of aggressive behavior are presented as a proportion of the cohort. **b** CBCL T-scores are presented against psychiatric diagnoses and/or behavioral concerns for each participant. Each row represents a participant. Scores were ranked by CBCL attention scores. CBCL attention and ADHD correlate with the presence of an ADHD diagnosis, but the same is not observed for the other CBCL subscales. * Denotes participants on psychotropic medications for the specified diagnosis. **c** BRIEF T-score subscales of FOXP1 participants (*n* = 17) presented against normed samples for ASD, ADHD-combined type, and individuals with typical development

The most common behavioral comorbidity observed in our cohort was ADHD; 15/21(71%) participants received a consensus diagnosis of ADHD, with 13 meeting DSM-5 diagnostic criteria for ADHD-combined presentation and two for predominantly inattentive presentation. In addition, anxiety (10/21, 47%) and aggressive behavior (9/21, 42%) were reported and/or observed in a noteworthy proportion of individuals (Fig. 5a). Aggressive behavior (verbal or physical) was almost exclusively described in the context of poor frustration tolerance; however, when present it was frequent enough that caregivers considered it interfered with daily functioning. Importantly, empirically based scales did not always fully capture the extent of behavioral symptoms observed by clinicians and reported verbally by caregivers during the evaluation. ADHD scores on the CBCL/ABCL performed better than anxiety scores; however, the instrument did not fully capture the extent of behavioral and psychiatric symptoms (Fig. 5b). No other concerning behavioral symptoms were identified in our cohort; scores on the other CBCL/ABCL scales (e.g., Depressive) fell in the normal range (*T* score < 65). Additional file 2: Table S2 summarizes the most salient results for our cohort. In terms of executive functioning, participants performed worse on the inhibition and working memory scales as a group, with mean scores approaching those observed in a normative ADHD-combined type population (Fig. 5c). Interestingly, scores on the emotional control scale were similar to those observed in typically developing children. None of the scores differed significantly from those reported for normed ASD and ADHD-combined samples (all *p* > 0.5).

### Medical evaluation

Frequent (> 50%) medical comorbidities are shown in Table 2; all medical comorbidities including common (20–50%) and less commonly reported findings (< 20%) are shown in Additional file 3: Table S3 and Additional file 4: Table S4.

**Table 2:** Medical findings

Feature	Total cohort (%)
Hypotonia	95
Wide nasal bridge	95
Bulbous nose	90
Ocular abnormality	77
*Strabismus*	45
*Nystagmus*	14
*Amblyopia*	14
Recurrent infections	77
*Otitis media*	50
*Upper respiratory infection*	41
*Urinary tract infection*	9
Gait abnormalities	67
Malocclusions	63
Hypoplastic/dysplastic nails	55
Feeding issues	55
High arched palate	53

Frequent (> 50%) and common (20–50%) medical findings are listed (*n* = 22)

**Fig. 6:**
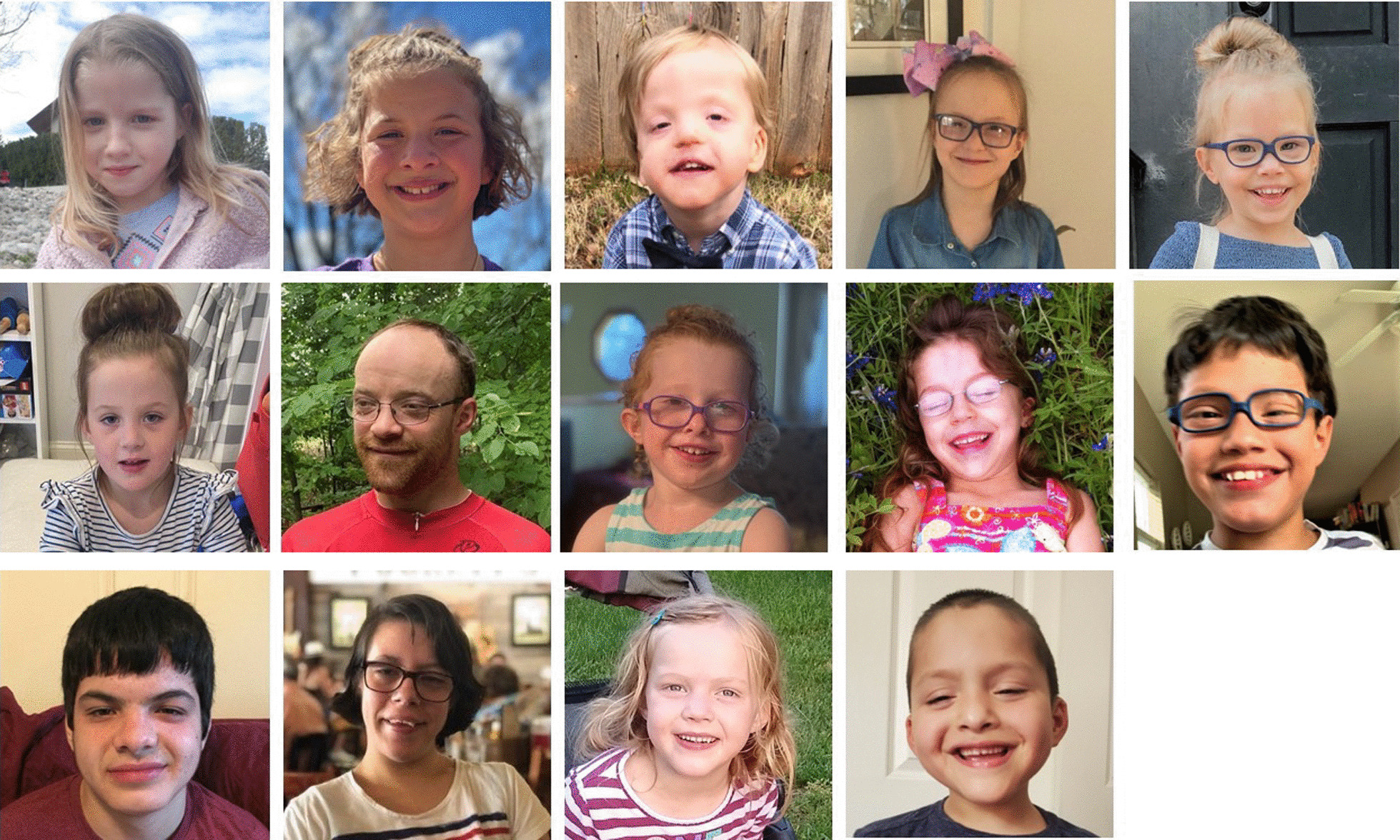
Pictures of participants

The most common medical finding was hypotonia, present in 21 out of 22 individuals. Ocular abnormalities followed (17/22); of the ocular abnormalities the most common findings were strabismus (10/22), esotropia (6/22), nystagmus (3/22), and amblyopia (3/22). Recurrent infections were present in 17/22 individuals. Otitis media was the most common recurrent infection (11/22), followed by respiratory tract infections (9/22), urinary tract infections (2/22), and skin infections (1/22). Gait abnormalities were observed in 12 out of 22 participants. Lastly, feeding issues were present in approximately half of the sample (12/22). Common medical comorbidities include gastrointestinal problems (9/22), sleep disturbance (7/22), neonatal problems (7/22), ptosis (6/22), and structural brain abnormalities (5/19). Of the gastrointestinal problems, the most common finding was reflux (6/20). Of the neonatal problems, 4/22 individuals required a neonatal intensive care unit (NICU) stay, 3/22 reported meconium aspiration, and 2/22 reported hyperbilirubinemia. Structural brain anomalies were present in 5 individuals out of the 19 who had reported completing an MRI. These included two individuals with periventricular leukomalacia, and one individual each with prominent lateral ventricles, mild thickening of the corpus callosum, hypoplastic vermis, and Chiari malformation type 1 (Fig. 6).

Less commonly reported medical comorbidities include congenital heart defects (3/22), seizures (3/22), hearing problems (2/22), and obstructive sleep apnea (1/22). Congenital heart defects were present in three participants, characterized by (1) patent ductus arteriosus and an atrial septal defect, (2) atrial septal defect and a patent foramen ovale, and (3) atrial septal defect, pulmonary stenosis, enlarged right ventricle, deformed pulmonary valve, and dilated main pulmonary artery. This latter individual carried a pathogenic variant in KCNQ1. Clinical neurological evaluation further described hypotonia, gait abnormalities, and dysarthria in all participants (Additional file 5: Table S5).

## Discussion

The present study provides in-depth characterization of the neurodevelopmental, behavioral, and medical profile in a prospective single-center cohort of 22 individuals with FOXP1 syndrome. Our findings reinforce the neurobehavioral phenotype of FOXP1 syndrome as a condition characterized by mild to moderate intellectual disability, motor delays, language impairment, and a range of behavioral features that are characteristic of ASD, ADHD, and anxiety disorders. We found that a diagnosis of ADHD is warranted in the vast majority of individuals with FOXP1 syndrome. In addition, clinicians and parents reported high levels of anxiety, although they were poorly captured with empirical instruments. Remarkably, a significant proportion of individuals with ADHD or anxiety diagnoses were not receiving associated psychopharmacological or behavioral interventions, indicating that there may be immediate opportunities for improved care and quality of life for some individuals with FOXP1 syndrome and their families.

The genetic landscape across participants in our sample is comparable to previous samples and includes a combination of missense variants clustered in the forkhead DNA-binding domain, protein truncating variants across the gene, and partial or full deletions of the *FOXP1* gene (Fig. 1). The high frequency of missense variants in the DNA-binding surface and domain swapping region critical for FOXP1-dimerization aligns with previous observations [11, 56].

Consistent with prior studies, we found that 76% (13/17 over age five) of individuals in our cohort had ID, with the majority falling in the mild to moderate ID range. One individual’s IQ scores, who was previously described by Siper et al. [11], fell in the average range. However, in contrast to our previous observation, we didn’t observe a correlation between age and performance on cognitive testing or adaptive functioning. This reinforces the notion that individuals with FOXP1 syndrome present with a range of cognitive abilities. Furthermore, we observed some discrepancies in terms of adaptive functioning and IQ scores. For example, the participant with a FSIQ of 93 (VIQ 92, NVIQ 95) scored 69 on the Vineland Adaptive Behavior Composite. Similarly, the participant with the highest adaptive behavior scores (Vineland-3 Adaptive Composite standard score of 80) had a FSIQ of 50 (VIQ 56, NVIQ 44). This observation aligns with the notion that adaptive functioning is influenced by a number of factors, including the presence of associated behavioral symptoms and/or response and availability of therapeutic interventions.

Assessment of adaptive functioning showed a tendency for comparatively higher socialization skills than daily living or communication abilities (Figs. 2, 3). Motor milestones were uniformly delayed, with participants taking on average, close to double the amount of time to develop skills, such as crawling (15 months) and walking independently (22 months), compared to typical peers. All individuals at the time of participation walked independently, with the exception of the youngest participant, who has since achieved this skill, indicating that this important milestone is likely to be universally achieved. Fine motor and gross motor skills, as measured by the Vineland-3, did not show significant differences to those previously described. Clinician-administered evaluations found that 25% of participants had impaired visual-motor integration, and parent report indicated that 100% of participants had a developmental coordination disorder. In support of this, the neurological examination found abnormal gait in 38% of individuals at time of examination. Broadly, neurological and medical findings were consistent with prior reports. Interestingly, many medical features could be related to atypical motor and muscle development (e.g., hypotonia, impaired visuo-motor integration, gait abnormalities, or gastrointestinal dysfunction). Indeed, an animal study with Foxp1+/− mice reflecting FOXP1 haploinsufficiency found pronounced muscular atrophy in the esophagus and colon, caused by reduced muscle cell proliferation and associated dysregulation of genes implicated in smooth and skeletal muscle morphogenesis and function [57].

Language and communication varied widely across participants, with some individuals scoring three standard deviations below normative scores and one individual presenting with nearly typical receptive and expressive language ability (Fig. 3). Importantly, the only participant in our cohort that did not have language at the time of evaluation was 25 months, suggesting, as has been previously reported, that individuals with FOXP1 syndrome are likely to develop language abilities, albeit at a slower pace than their typically developing peers [11, 58]. Contrary to what had been previously reported, we found no difference between expressive and receptive language abilities. A recent prospective study by Braden et al. [29] describes language deficits in FOXP1 as a speech production disorder characterized by dysarthria, poor motor planning with high frequency of phonologic errors. Interestingly, they also found better expressive than receptive language abilities, but only in individuals with loss of function variants. Nonetheless, the relatively small sample size of all cohorts to date, and conflicting findings in regards to receptive versus expressive language, prevents us from drawing definitive conclusions. Another important observation in the current study was the overall better performance in direct norm-reference language assessments (i.e., PPVT-4 and EVT-2) than captured by the Vineland-3, which relies on caregiver report and is reflective of everyday function. This observation suggests that the complex neurobehavioral profile in FOXP1 syndrome is likely to impact an individual’s ability to effectively and independently utilize language in everyday life situations, impacting their ability to communicate. Taken in conjunction with findings from prior studies, our results support the central role that speech and language deficits play in the phenotype of FOXP1 syndrome and emphasize the need to introduce speech therapy early in development.

Based on previous reports, we paid careful attention to ASD symptomology. The majority of individuals (~ 60%) fell above cutoff scores on standardized instruments. However, only 24% of participants met criteria for a consensus diagnosis of ASD. Although a large number of participants (95%) presented with difficulties in developing and maintaining friendships, a smaller subgroup presented with core deficits in social communication and interactions (i.e., socio-emotional reciprocity and nonverbal communication) (Additional file 6: Table S6), central to the diagnosis. Repetitive behaviors were almost universally present in our cohort (95%) and were not restricted to a diagnosis of ASD. Sensory symptoms, in particular sensory seeking behaviors across visual, tactile, and auditory domains, were more common than hyperreactivity and hyporeactivity symptoms in our sample. Four of the five individuals that met ASD criteria by expert clinical consensus received the Module 1 (60%) or Module 2 (20%), suggesting limited language abilities in this group. Four out of the five participants with ASD were females, which is in line with the larger proportion of females in our sample and the overall notion that there is no sex difference in the prevalence of ASD in FOXP1 Syndrome.

Further exploratory analysis found that mean IQ, PPVT-4, and EVT-2 scores were significantly lower for the ASD group [*F* (1,19) = 2.73, *p* = 0.041)]. Although these observations could suggest that ASD is associated with a more severe phenotype, there are multiple confounding factors that prevent us from drawing conclusions. Among them, the small sample size and proportion of individuals with ASD and the wide age range of participants. Importantly, these difference in norm-referenced measures did not extend to statistically significant differences in adaptive behavior scores between groups. This latter observation emphasizes the need for larger longitudinal studies adequately powered to evaluate the impact of ASD and comorbid behavioral symptoms on daily functioning of individuals with FOXP1 syndrome.

Overall, standardized measures of ASD symptoms in this sample showed high sensitivity, but low specificity. Indeed, all participants in the ASD consensus group met cutoff scores on ASD clinician-administered and caregiver-reported instruments (Fig. 4, Additional file 6: Table S6). Nonetheless, several others also met criteria for ASD on gold-standard instruments when clinical consensus rejected a true ASD diagnosis. These findings are not unique to our study and mirror other reports in ID and in genetic neurodevelopmental syndromes [59–64]. Furthermore, they highlight the limitations of these instruments when evaluating individuals with complex neurobehavioral profiles including those with high rates of externalizing behaviors, like ADHD symptoms [65]. Finally, this observation underscores the challenge clinicians often face when evaluating individuals such as those in our cohort and emphasizes the value of multidisciplinary teams and the importance of utilizing several methods for diagnostic assessment and interpreting results carefully.

Psychiatric comorbidities were commonly noted by clinicians and a prevalent area of concern for caregivers. Consensus diagnosis of ADHD, based on caregiver report, direct observation by expert clinicians and results from empirical instruments, was assigned to 71% of participants. Furthermore, anxiety symptoms were observed in 47% of participants, and concerns for aggressive behavior were reported in 42% of participants. However, CBCL subscales only partially captured these observations, which may suggest the need to apply different tools to quantify psychiatric symptoms in populations such as this.

Clarifying the nature and prevalence of psychiatric symptoms stands to have important implications for treatment and overall quality of life. For example, aggressive behavior can reflect a mediative response in children with anxiety disorders and/or ADHD symptoms, in particular in the presence of ID and language impairments [66]. Furthermore, both anxiety and ADHD symptoms often respond to psychopharmacological interventions. It is noteworthy that only 23% (5/21: 3 for ADHD, 1 for ADHD and anxiety, and 1 for anxiety) of individuals in our sample were being treated for ADHD and/or anxiety symptoms, suggesting that these conditions are under-recognized and undertreated by community providers treating individuals with FOXP1 syndrome.

## Limitations

Given our small sample size and limitations in the interpretation of missense variants without functional studies of the protein, we are unable to report genotype–phenotype associations. This limitation in the sample size is compounded by the wide age range of participants, hampering our ability to draw conclusions on symptom variability across the lifespan. Furthermore, study participants came from across the USA and Canada, where service availability can vary significantly by geographical location, which makes it difficult to systematically report on current educational settings, service utilization, and therapeutic interventions. Finally, our study did not include academic achievement testing, which could strengthen sample characterization and support the development of educational and additional therapeutic recommendations.

## Conclusions

Individuals with FOXP1 syndrome present with a complex and variable neurobehavioral profile characterized by mild to moderate intellectual disability, speech delays, motor impairments and high rates of neurobehavioral symptoms, which include ASD features, ADHD, and anxiety disorders. Our findings also highlight the inherent limitations of standardized instruments when it comes to assessing individuals with complex neurobehavioral profiles and underscore the importance of clinical judgment when interpreting results. Consistent with previous studies, aggressive behavior was observed in a proportion of individuals; however, we conclude that aggression appeared to be secondary to poorly treated ADHD and anxiety symptoms compounded by limited language abilities. Finally, our observations offer an opportunity for immediate intervention as both ADHD and anxiety symptoms can respond to available evidence-based pharmacological and behavioral treatments that could improve long-term outcomes.

## Supplementary Information

**Additional file 1. Supplemntal Table 1:** Genetic variants.

**Additional file 2. Supplemental Table 2:** Neuropsychological assessments.

**Additional file 3. Supplemental Table 3:** Medical comorbidities.

**Additional file 4. Supplemental Table 4:** Dysmorphic features in the cohort.

**Additional file 5. Supplemental Table 5:** Neurological exam.

**Additional file 6. Supplemental Table 6:** Autism spectrum disorder symptoms.

## Data Availability

The majority of the dataset used during the current study is included in this published article and supplementary information file. The remainder of the dataset is available from the corresponding author on reasonable request and subsequent ethics review.

## Abbreviations

ABCL: Adult Behavior Checklist
ADHD: Attention-deficit/hyperactivity disorder
ADI: Autism Diagnostic Interview
ADI-R: Autism Diagnostic Interview—Revised
ADOS-2: Autism Diagnostic Observation Schedule, Second Edition
ASD: Autism spectrum disorder
BRIEF-2: Behavior Rating Inventory of Executive Function
CBCL: Child and Adult Behavior Checklist
CLIA: Clinical Laboratory Improvement Amendments
DAS-II: Differential Ability Scales—Second Edition
DCD: Developmental coordination disorder
DCDQ: Developmental Coordination Disorder Questionnaire 2007
DQ: Developmental quotient
DSM-5: Diagnostic and Statistical Manual—5th Edition
EEG: Electroencephalogram
EVT-2: Expressive Vocabulary Test, 2nd Edition
FOXP1: Forkhead box P1, OMIM 605515
HGVS: Human Genome Variation Society
ID: Intellectual disability
IQ: Intellectual quotient
MIM: Mendelian inheritance in man
MRI: Magnetic resonance imaging
NDD: Neurodevelopmental disorder
NICU: Neonatal intensive care unit
PPVT-4: Peabody Picture Vocabulary Test, 4th Edition
RBS-R: Repetitive Behavior Scale—Revised
SAND: Sensory assessment for neurodevelopmental disorders
SRS-2: Social Responsiveness Scale, 2nd Edition
SSP: Short Sensory Profile
TD: Typically developing
VABS-II: Vineland Adaptive Behavior Scales—Second Edition
Vineland-3: Vineland Adaptive Behavior Scales—Third Edition
VMI-6: Beery–Buktenica Developmental Test of Visual-Motor Integration, Sixth Edition
