# Supplementary material for: Individuals with FOXP1 syndrome present with a complex neurobehavioral profile with high rates of ADHD, anxiety, repetitive behaviors, and sensory symptoms
Source: Mol Autism. 2021 Sep 29;12:61. doi: 10.1186/s13229-021-00469-z (PMC8482569; doi:10.1186/s13229-021-00469-z)
Supplement: Supplementary file 1 — Additional file 1. Supplemntal Table 1: Genetic variants. [file 13229_2021_469_MOESM1_ESM.pdf]

Supplemntal Table 1: Genetic variants

ID	Sex	Coding DNA change[1]	Protein change	Effect	Inheritance	Classification
S1	M	c.1393A>G	p.Arg465Gly	Missense	*De Novo*	Pathogenic
S2	F	c.1267_1268delGT	p.Val423Hisfs*37	Frameshift	*De Novo*	Pathogenic
S2	F	c.1333_1335delinsAA	p.Val445Asnfs*29	Frameshift	*De Novo*	Pathogenic
S4	F	c.1506C>G	p.Phe502Leu	Missense	*De Novo*	Likely Pathogenic[b]
S5	F	c.975-2A>C	p.Lys325Asnfs*12	Splice Site	*De Novo*	Pathogenic
S6	M	c.1481G>A	p.Trp494*	Nonsense	*De Novo*	Pathogenic
S7	F	c.1538T>C	p.Val513Ala	Missense	*De Novo*	Likely Pathogenic
S8	F	c.1507C>T	p.Arg503*	Nonsense	*De Novo*	Pathogenic
S9	F	c.1240_1241delCT	p.Leu414Aspfs*46	Frameshift	*De Novo*	Pathogenic
S10	F	c.1630C>T	p.Arg544*	Nonsense	*De Novo*	Pathogenic
S11	M	c.1574G>A	p.Arg525Gln	Missense	Not maternally inherited [a]	Pathogenic
S12	F	c.1541G>C	p.Arg514Pro	Missense	*De Novo*	Pathogenic
S15	M	c.1240dupC	p.Leu414Profs*47	Frameshift	Not maternally inherited [a]	Pathogenic
S16	F	c.1489C>T	p.Arg497*	Nonsense	*De Novo*	Pathogenic
S17	F	c.1103dup	p.His368Glnfs*93	Frameshift	*De Novo*	Likely Pathogenic
S18	F	c.1541G>A	p.Arg514His	Missense	*De Novo*	Pathogenic
S19	F	c.529C>T	p.Gln177*	Nonsense	*De Novo*	Pathogenic
S20	F	c.1541G>A	p.Arg514His	Missense	*De Novo*	Pathogenic
S21	F	c.822delT	p.His274Glnfs*51	Frameshift	Not maternally inherited [a]	Pathogenic
S22	M	c.1889+5G>T	IVS20+5G>T	Splice Site	*De Novo*	Likely Pathogenic
ID	Sex	Array result[2]	Deletion size	Effect	Inheritance	Classification
S13	M	arr3p13p12.3(70765385-79219598)x1	8500 Kb	Deletion	Unknown	Abnormal
S14	M	arr3p13(71080759-71148304) x1	67.5 Kb	Partial deletion	*De Novo*	Abnormal

[1]Coding transcript used: NM_032682.5

[2]Assembly used: hg19

[a]Father not available for testing

[b]Also carries a KCNQ1 pathogenic variant
