# Supplementary material for: Individuals with FOXP1 syndrome present with a complex neurobehavioral profile with high rates of ADHD, anxiety, repetitive behaviors, and sensory symptoms
Source: Mol Autism. 2021 Sep 29;12:61. doi: 10.1186/s13229-021-00469-z (PMC8482569; doi:10.1186/s13229-021-00469-z)
Supplement: Supplementary file 2 — Additional file 2. Supplemental Table 2: Neuropsychological assessments. [file 13229_2021_469_MOESM2_ESM.pdf]

Supplemental Table 2: Neuropsychological assessments

		Cohort 1 (n=5)				Cohort 2 (n=17)				Total (n=22)			
Domain	Variable	n	Mean	St. Dev	% atypical score	n	Mean	St. Dev	% atypical score	n	Mean	St. Dev	% atypical score
Intellectual and adaptive functioning	Full scale IQ	Stanford Binet-5, n=5	61.80	19.33	80%	Stanford Binet-5, n=10 Mullen Scales, n=5 DAS-II, n=2	53.21	18.97	88%	22	55.16	18.94	86%
	Nonverbal IQ		65.80	19.36	80%		59.35	19.73	82%		60.82	19.38	82%
	Verbal IQ		60.80	18.46	80%		52.54	21.06	82%		54.42	20.38	82%
	Vineland-3 Daily Living Skills	Did not complete Vineland-3	.	.	.	16, 1 completed the Vineland-2	57.13	13.66	94%	.	.	.	.
	Vineland-3 Socialization		.	.	.		60.19	16.72	88%	.	.	.	.
	Vineland-3 Adaptive Behavior Composite		.	.	.		54.63	13.86	88%	.	.	.	.
Receptive and expressive language	Vineland-3 Communication	Did not complete Vineland-3	.	.	.	16, 1 completed the Vineland-2	44.50	18.53	94%	.	.	.	.
	Vineland-3 Receptive		.	.	.		6.25	3.42	94%	.	.	.	.
	Vineland-3 Expressive		.	.	.		3.50	3.48	83%	.	.	.	.
	PPVT-4 standard score	5	67.00	17.89	80%	16, 1 did not complete due to functional capacity	62.94	20.53	76%	21	63.90	19.58	77%
	EVT-2 standard score	5	75.40	10.62	40%	14, 3 did not complete due to functional capacity	64.86	16.21	88%	19	67.63	15.41	77%
	MCDI Words Understood	Did not complete MCDI	.	.	.	17	310.12	123.10	.	.	.	.	.
	MCDI Words Produced		.	.	.		254.76	159.02	.	.	.	.	.
ASD symptomatology	ADOS-2 Social Affect	Module 2, n=2 Module 3, n=3	5.60	2.19	.	Module T, n=1 Module 1, n=4 Module 2, n=8 Module 3, n=2 Module 4, n=2	8.53	4.65	.	22	7.86	4.36	.
	ADOS-2 Restricted and Repetitive Behaviors		2.20	0.84	.		3.41	2.50	.		3.14	2.27	.
	ADOS-2 Comparison		4.20	1.79	.		5.33	1.99	.	20	5.05	1.96	57%
	ADI-R Socialization	5	16.40	5.94	100%	16, 1 too young for administration	11.88	7.01	50%	21	12.95	6.92	62%
	ADI-R Communication		11.00	4.36	80%		10.25	5.27	75%		10.43	4.98	76%
	ADI-R Restricted and Repetitive Behaviors		7.00	3.00	80%		4.06	2.57	68%		4.76	2.90	71%
	SRS-2 Social Awareness *T*-score	SRS-2 school age, n=5	74.80	4.97	100%	SRS-2 preschool, n=3 SRS-2 school age, n=13 SRS-2 adult, n=1	68.76	14.42	71%	22	70.14	13.03	77%
	SRS-2 Socal Cognitive *T*-score		75.20	9.20	100%		68.41	12.02	76%		69.95	11.61	82%
	SRS-2 Social Communication *T*-score		72.60	3.71	100%		64.00	14.09	59%		65.95	12.94	68%
	SRS-2 Social Motivation *T*-score		65.80	7.40	80%		53.76	13.64	24%		56.50	13.37	36%
	SRS-2 Restricted Interests and Repetitive Behavior *T*-score		75.00	10.10	100%		67.47	15.84	71%		69.18	14.86	77%
	SRS-2 Total *T*-score		75.20	5.36	100%		66.12	14.27	71%		68.18	13.26	77%
	SRS-2 Social Communication and Interaction *T*-score		74.60	4.77	100%		65.06	13.82	65%		67.23	12.91	73%
	SAND Total	5	20.40	10.83	.	17	28.82	11.90	.	22	26.91	11.97	.
	SAND Hyperreactivity		6.80	2.95	.		5.88	4.31	.		6.09	4.00	.
	SAND Hyporeactivity		4.60	2.41	.		6.24	4.55	.		5.86	4.17	.
	SAND Seeking		9.00	6.71	.		16.71	6.84	.		14.95	7.42	.
	SAND Visual		6.40	6.35	.		7.94	4.56	.		7.59	4.90	.
	SAND Tactile		7.00	3.81	.		12.12	4.76	.		10.95	4.98	.
	SAND Auditory		7.00	3.39	.		8.76	3.54	.		8.36	3.51	.
	RBS-R Stereotype Total	5	3.20	2.17	.	16	3.56	3.83	.	21	3.48	3.46	.
	RBS-R Self Injury Total		4.40	1.82	.		2.75	3.09	.		3.14	2.89	.
	RBS-R Compulsive Total		4.40	2.30	.		3.56	4.29	.		3.76	3.87	.
	RBS-R Ritualistic Total		2.60	2.41	.		2.63	3.03	.		2.62	2.84	.
	RBS-R Sameness Total		5.80	3.19	.		4.06	4.77	.		4.48	4.43	.
	RBS-R Restricted Total		4.00	2.55	.		2.56	2.42	.		2.90	2.47	.
	RBS-R Total		24.40	9.69	.		19.13	16.22	.		20.38	14.88	.
Behavioral comorbidities	ABC Irritability *T*-score	4	53.75	4.79	0%	15	45.47	5.83	0%	19	47.21	6.50	0%
	ABC Lethargy T-score		43.5	4.04	0%		41.67	2.18	0%		42.05	2.24	0%
	ABC Stereotypy T-score		48.75	8.5	0%		48.87	9.07	7%		46.47	8.80	5%
	ABC Hyperactivity T-score		55.5	9.15	25%		51.20	7.88	7%		52.11	8.09	11%
	ABC Speech T-score		44.25	2.98	0%		47.00	8.66	7%		46.42	7.82	5%
	CBCL Attention *T*-score	1.5-5y form, n=1 6-18y form, n=3	73	6.58	100%	1.5-5y form, n=6 6-18y form, n=10	69.88	9.20	81%	20	70.50	8.68	85%
	CBCL Aggressive *T*-score		68.25	2.36	100%		57.31	6.70	13%		59.50	7.51	30%
	CBCL Internalizing *T*-score		62.25	6.99	25%		48.25	9.38	0%		51.05	10.50	5%
	CBCL Externalizing *T*-score		69.25	1.71	100%		56.25	10.17	25%		58.85	10.15	40%
	CBCL Anxiety *T*-score		58.25	7.23	25%		54.63	5.68	6%		55.35	6.00	10%
	CBCL ADHD *T*-score		65	6.27	50%		64.63	7.25	69%		64.70	6.91	65%
	BRIEF Inhibit T-score	BRIEF-1, n=4	78	6.78	100%	BRIEF-1, n=4 BRIEF-Preschool, n=3 BRIEF-2, n=6, 1 particpant too old to complete	66.83	16.79	54%	17	69.63	15.52	65%
	BRIEF Shift T-score		76.5	11.33	100%		57.75	11.73	23%		62.44	14.03	41%
	BRIEF Emotional Control T-score		72.5	5.45	100%		49.75	10.70	8%		55.44	13.90	29%
	BRIEF Working Memory T-score		73	3.83	100%		66.25	12.71	54%		67.94	11.43	65%
	BRIEF Plan/Organize T-score		75	8.29	75%		52.58	14.15	23%		58.19	16.16	35%
Motor	VMI Standard	5	56.6	16.13	60%	16, 1 did not complete due to functional capacity	62.81	14.71	31%	21	61.22	14.89	38%
	DCDQ Fine Motor	4	7.75	0.96	.	16	6.25	2.27	.	20	6.55	2.14	.
	DCDQ Coordination		8.25	2.63	.		10.44	4.16	.		10.00	3.95	.
	DCDQ Total		27.75	5.91	100%		31.31	11.62	100%		30.60	10.69	100%

ABC	Aberrant Behavior Checklist- Community
ADI-R	Autism Diagnostic Interview-Revised
ADOS-2	Autism Diagnostic Observation Schedule, Second Edition
BEERY VMI	Beery-Buktenica Visual-Motor Integration
BRIEF-2	Behavior Rating Inventory of Executive Function, Second Edition
CBCL	Child Behavior Checklist
DCDQ	Developmental Coordination Disorder Questionnaire
EVT-2	Expressive Vocabulary Test
IQ	Intellectual quotient
MCDI	MacArthur Bates Communitative Development Index
PPVT-4	Peabody Picture Vocabulary Test
RBS-R	Repetitive Behaviors Scale - Revised
SAND	Sensory Assessment for Neurodevelopmental Disorders
SRS-2	Social Responsiveness Scale, 2nd Edition
