# Supplementary material for: Individuals with FOXP1 syndrome present with a complex neurobehavioral profile with high rates of ADHD, anxiety, repetitive behaviors, and sensory symptoms
Source: Mol Autism. 2021 Sep 29;12:61. doi: 10.1186/s13229-021-00469-z (PMC8482569; doi:10.1186/s13229-021-00469-z)
Supplement: Supplementary file 3 — Additional file 3. Supplemental Table 3: Medical comorbidities. [file 13229_2021_469_MOESM3_ESM.pdf]

Supplemental Table 3. Medical comorbidities											
	Cohort 1	Cohort 2	Total Cohort	S1	S2	S3	S4	S5	S6	S7	S8
Structural brain change	40%	19%	24%	1	0	0	0	1	0	0	0
Seizures	0%	18%	14%	0	0	0	0	0	0	1	0
Ocular abnormality	60%	82%	77%	0	1	1	1	0	1	1	1
Strabismus	60%	41%	45%	0	1	1	1	0	1	0	1
Esotropia	0%	35%	27%	0	0	0	0	0	0	1	0
Nystagmus	0%	18%	14%	0	0	0	0	0	0	0	0
Amblyopia	0%	18%	14%	0	0	0	0	0	0	1	0
Hearing problems	0%	12%	9%	0	0	0	0	0	0	0	0
Hypotonia	100%	94%	95%	1	1	1	1	1	1	0	1
Sleep disturbance	0%	41%	32%	0	0	0	0	0	0	1	0
Obstructive sleep apnea	0%	6%	5%	0	0	0	0	0	0	0	0
Feeding issues	20%	65%	55%	1	0	0	0	0	0	0	0
Recurrent infections	60%	82%	77%	1	0	1	1	0	1	0	1
Otitis media	60%	47%	50%	1	0	1	1	0	1	0	0
Upper respiratory infection	20%	47%	41%	0	0	1	0	0	0	0	1
Urinary tract infection	20%	6%	9%	0	0	1	0	0	0	0	0
Skin infection	20%	0%	5%	1	0	0	0	0	0	0	0
Congenital heart defect	20%	12%	14%	0	0	0	1	0	0	0	0
Gastrointestinal abnormaliti	20%	47%	41%	0	0	0	0	1	0	0	0
Reflux	0%	38%	30%	0	0	0	0	0	0	0	0
Constipation	20%	0%	5%	0	0	0	0	1	0	0	0
Gait abnormalities	100%	56%	67%	1	1	1	1	1	1	1	1
Neonatal problem	40%	29%	32%	0	0	1	0	1	0	0	0
Required NICU stay	40%	12%	18%	0	0	1	0	1	0	0	0
Meconium aspiration	20%	12%	14%	0	0	1	0	0	0	0	0
Hyperbilirubinemia	20%	6%	9%	0	0	0	0	1	0	0	0

S9	S10	S11	S12	S13	S14	S15	S16	S17	S18	S19	S20	S21	S22
1	0	0	0	0	0	0	0	0	1	1	0	0	n/a
0	1	0	1	0	0	0	0	0	0	0	0	0	0
1	1	0	1	1	1	1	1	1	1	0	1	0	1
0	0	0	1	1	0	1	0	1	0	0	0	0	1
0	1	0	0	1	1	0	1	0	0	0	1	0	0
0	0	0	0	0	0	1	0	1	1	0	0	0	0
0	0	0	0	0	1	0	1	0	0	0	0	0	0
0	0	0	0	1	0	0	0	0	0	1	0	0	0
1	1	1	1	1	1	1	1	1	1	1	1	1	1
0	1	1	0	0	1	1	0	0	0	1	0	1	0
0	0	0	0	0	1	0	0	0	0	0	0	0	0
1	0	1	1	1	1	1	1	1	1	0	1	1	0
1	1	1	1	1	1	0	1	1	0	1	1	1	1
1	1	1	1	1	0	0	0	1	0	1	0	0	0
0	0	1	0	0	1	0	1	1	0	1	0	1	1
0	0	0	0	0	0	0	0	0	0	0	1	0	0
0	0	0	0	0	0	0	0	0	0	0	0	0	0
0	0	0	0	1	0	0	0	0	0	1	0	0	0
0	0	0	1	1	0	0	1	1	1	1	1	1	0
0	0	0	1	n/a	0	0	1	1	n/a	1	1	1	0
0	0	0	0	n/a	0	0	0	0	n/a	0	0	0	0
1	0	0	1	n/a	0	1	0	1	1	0	1	0	0
1	0	0	0	1	0	0	0	0	1	0	1	1	0
1	0	0	0	0	0	0	0	0	1	0	0	0	0
1	0	0	0	1	0	0	0	0	0	0	0	0	0
0	0	0	0	0	0	0	0	0	0	0	1	0	0
