# Supplementary material for: Individuals with FOXP1 syndrome present with a complex neurobehavioral profile with high rates of ADHD, anxiety, repetitive behaviors, and sensory symptoms
Source: Mol Autism. 2021 Sep 29;12:61. doi: 10.1186/s13229-021-00469-z (PMC8482569; doi:10.1186/s13229-021-00469-z)
Supplement: Supplementary file 4 — Additional file 4. Supplemental Table 4: Dysmorphic features in the cohort. [file 13229_2021_469_MOESM4_ESM.pdf]

Supplemental Table 4: Dysmorphic features in the cohort

	Cohort 1	Cohort 2	Total Cohort
**Head / Brain / Face**			
Macrocephaly	67%	43%	47%
Microcephaly	0%	0%	0%
Dolicocephaly	25%	21%	22%
Micrognathia	0%	7%	5%
Full cheeks	60%	33%	40%
Malar hypoplasia	0%	53%	40%
Flat midface	20%	53%	45%
Pointed chin	20%	33%	30%
Wide nasal bridge	100%	93%	95%
Bulbous nose	80%	93%	90%
Long philtrum	60%	27%	35%
**Eyes**			
Periorbital fullness	40%	29%	32%
Epicanthal folds	20%	7%	11%
Ptosis	20%	36%	32%
Deep set eyes	20%	14%	16%
Long eyelashes	20%	23%	22%
Hypertelorism	50%	10%	21%
**Ears**			
Low set ears	0%	0%	0%
Ear anomalies	20%	14%	16%
**Mouth**			
Full lips	60%	13%	25%
High arched palate	80%	43%	53%
Malocclusions	60%	64%	63%
**Neck / Back**			
Hyperextensiblity	20%	40%	35%
Abnormal spine curvature	20%	0%	5%
Sacral dimple	20%	33%	30%
Short neck	20%	7%	10%
**Skin / Hair**			
Sparse hair/abnromal hair whorl	20%	20%	20%
**Hands / Feet**			
Large/fleshy hands	0%	7%	5%
5th finger clinodactyly	40%	33%	35%
Hypoplastic / dysplasic nails	20%	67%	55%
Syndactlyly of toes 2 and 3	20%	0%	5%
