# Supplementary material for: Individuals with FOXP1 syndrome present with a complex neurobehavioral profile with high rates of ADHD, anxiety, repetitive behaviors, and sensory symptoms
Source: Mol Autism. 2021 Sep 29;12:61. doi: 10.1186/s13229-021-00469-z (PMC8482569; doi:10.1186/s13229-021-00469-z)
Supplement: Supplementary file 5 — Additional file 5. Supplemental Table 5: Neurological exam. [file 13229_2021_469_MOESM5_ESM.pdf]

Supplemental Table 5: Neurological exam

	S1	S3	S4	S5	S6	S7	S8	S9	S10	S11
Hypotonia	1	1	1	1	1	0	1	1	1	0
Hypotonia	Mild	Mild	Mild	Mild	Mild		Moderate	Mild, upper extremeties	Mild	
Sensory Exam (touch, mild pain)		Symmetrical response to touch and mild pain	Symmetrical response to touch and mild pain	Symmetrical response to touch and mild pain	Symmetrical response to touch and mild pain	Symmetrical response to touch and mild pain	Symmetrical response to touch and mild pain	Symmetrical response to touch and mild pain	Symmetrical response to touch and mild pain	Symmetrical response to touch and mild pain
Gait abnormal	1	1	1	1	1	0	0	1	0	0
Gait Note	Gait was mildly abnormal, mostly due to axial, and some limb hypotonia.	Gait is mildly abnormal with mild hypotonia, reduced upper extremity associated movements, and external feet deviation.	Mildly abnormal, hypotonic	Gait was mildly abnormal because of bilateral internal deviation of feet while walking and minimally broad base when running	Gait was mildly abnormal with mild toe-walking, disorganization, difficulties walking stairs			Gait was moderately abnormal with flexed knees and toe walking		
Dysarthria	0	1	0	0	0	0	0	0	0	1
Other Notes	Motor coordination abnormalities, mildly abnormal posture, delayed speech/language for age, decreased relatedness and attention span	Motor coordination and visual motor coordination abnormalities, stuttering speech	Fine motor clumsiness, speech and language delay	Does not yet have bowel and bladder control.	Abnormal motor coordination and mildly brisk deep tendon reflexes	Mild ptosis right>left sided	Motor coordination abnormalities. Expressive and receptive speech/language disorder, hyperactivity and impulsivity	Macrocephaly, brisk deep tendon reflexes in lower extremities with clonus, abnormal motor coordination, short attention span	Fine motor clumsiness, and abnormal speech	Spina bifida of lumbar region without hydrocephalus reduced language performance, incontinence unrelated to spine abnormalities

S13	S14	S15	S16	S17	S18	S19	S20	S21	S22
1	1	1	1	1	1	1	1	1	0
Moderate	Mild-moderate,	Mild, truncal	Mild	Mild	Mild	Mild, truncal	Mild	Mild	
Symmetrical response to touch and mild pain	Symmetrical response to touch and mild pain	Symmetrical response to touch and mild pain	Symmetrical response to touch and mild pain	Symmetrical response to touch and mild pain	Symmetrical response to touch and mild pain	Symmetrical response to touch and mild pain	Symmetrical response to touch and mild pain	Symmetrical response to touch and mild pain	Symmetrical response to touch and mild pain
1	n/a	0	1	0	1	0	1	0	0
Gait was ataxic with intermittent toe-walking, feet turned out; Upper extremities held in a "guarding" position; needed help to go up and downstairs.	Does not walk yet		Gait was disorganized with a mildly wider base		Gait showed intermittent toe-walking		Gait was abnormal and disorganized; No coordinated UE/LE movements, occasionally on toes, feet pronated, knees flexed.		
0	0	1	0	0	0	0	1	1	0
Expressive and receptive speech/language disorder, short attention span	Dysphagia (with a strong gag reflex), reduced facial movements, difficulties producing sounds, motor coordination abnormalities	Reduced motor coordination and inattention	Very short attention span, hyperactivity, anxiety, aggression, abnormal language development, sleep abnormality, small head circumference	Mild limitation of vertical upward gaze, reduced visual motor coordination	Expressive and receptive language disorders, a short attention span	Fine motor coordination abnormalities, short attention span and reduced visual tracking	Motor coordination abnormalities, expressive and receptive language disorders, short attention span with significant hyperactivity and impulsivity, and mild drooling	Macrocephaly, multiple skin flat red areas (possibly hemangiomas), short attention span	Mild motor coordination abnormalities, mild articulation problems, reportedly reduced vision in the left eye, nystagmus, looking left and vertical gaze abnormalities (Rt eye goes up with bilateral conversion)
