# Supplementary material for: Individuals with FOXP1 syndrome present with a complex neurobehavioral profile with high rates of ADHD, anxiety, repetitive behaviors, and sensory symptoms
Source: Mol Autism. 2021 Sep 29;12:61. doi: 10.1186/s13229-021-00469-z (PMC8482569; doi:10.1186/s13229-021-00469-z)
Supplement: Supplementary file 6 — Additional file 6. Supplemental Table 6: Autism spectrum disorder symptoms. [file 13229_2021_469_MOESM6_ESM.pdf]

Supplemental Table 6: Autism spectrum disorder symptoms

<table>
<tr><th>Assessment</th><th colspan="5">Classification</th></tr>
<tr><th>Domain</th><th colspan="2">Description; range; M (SD)</th><th colspan="2">Met criteria; n (%)</th><th>ASD Diagnosis/Met classification; n(%)</th></tr>
<tr><td colspan="6">ASD Consensus</td></tr>
<tr><td></td><td colspan="4">Based on ADOS-2, ADI and clinical evaluation (DSM-5)</td><td>5 (24)</td></tr>
<tr><td colspan="6">Psychiatric Evaluation</td></tr>
<tr><td rowspan="3">Social communication and interaction deficits (n=21)</td><td colspan="2">Deficits in socio-emotional reciprocity</td><td>8 (38)</td><td rowspan="3">5 (35)</td><td rowspan="7">5(24)</td></tr>
<tr><td colspan="2">Deficits in non-verbal communication</td><td>5 (24)</td></tr>
<tr><td colspan="2">Deficits in developing and maintaining friendships</td><td>20 (95)</td></tr>
<tr><td rowspan="4">Repetitive Behaviors (n=11)</td><td colspan="2">Stereotyped behaviors</td><td>17 (80)</td><td rowspan="4">20(95)</td></tr>
<tr><td colspan="2">Insistence of sameness</td><td>15 (71)</td></tr>
<tr><td colspan="2">Highly restricted interests</td><td>16 (76)</td></tr>
<tr><td colspan="2">Sensory symptoms</td><td>20 (95)</td></tr>
<tr><td colspan="6">ADOS-2</td></tr>
<tr><td rowspan="3">Social affect calibrated scores (n=19)</td><td>1-3</td><td rowspan="3">5.1(1.77)</td><td colspan="2">5 (26)</td><td rowspan="9">12(57)</td></tr>
<tr><td>4-5</td><td colspan="2">7 (37)</td></tr>
<tr><td>6-10</td><td colspan="2">7 (37)</td></tr>
<tr><td rowspan="3">Repetitive behavior calibrated score (n=19)</td><td>1-3</td><td rowspan="3">6.33(2.52)</td><td colspan="2">2 (11)</td></tr>
<tr><td>4-5</td><td colspan="2">4 (21)</td></tr>
<tr><td>6-10</td><td colspan="2">13 (68)</td></tr>
<tr><td rowspan="3">ADOS comparison scores (n=21)</td><td>No ASD (1-3)</td><td rowspan="3">5.25 (1.94)</td><td colspan="2">9 (43)</td></tr>
<tr><td>ASD (4-5)</td><td colspan="2">2 (10)</td></tr>
<tr><td>Autism (6-10)</td><td colspan="2">10 (47)</td></tr>
<tr><td colspan="6">ADI-R</td></tr>
<tr><td>Socialization (n=21)</td><td>3 – 26</td><td>13.1 (7.06)</td><td colspan="2">13 (61)</td><td rowspan="3">10(47)</td></tr>
<tr><td>Communication (n=21)</td><td>2 – 21</td><td>10.6 (5.04)</td><td colspan="2">16 (76)</td></tr>
<tr><td>Repetitive Behaviors (n=21)</td><td>0 – 9</td><td>4.6 (2.87)</td><td colspan="2">15 (71)</td></tr>
</table>
